# Tissue-Specific Immune Transcriptional Signatures in the Bordering Tissues of the Mouse Retina and Brain

**DOI:** 10.1167/iovs.65.12.42

**Published:** 2024-10-28

**Authors:** Fazeleh Etebar, Paul Whatmore, Damien G. Harkin, Samantha J. Dando

**Affiliations:** 1 Centre for Immunology and Infection Control, School of Biomedical Sciences, Faculty of Health, Queensland University of Technology, Brisbane, Australia; 2 eResearch Office, Research Infrastructure, Queensland University of Technology, Brisbane, Australia; 3 Centre for Vision and Eye Research, School of Biomedical Sciences, Faculty of Health, Queensland University of Technology, Brisbane, Australia

**Keywords:** choroid, leptomeninges, immune cells, single cell RNA sequencing

## Abstract

**Purpose:**

Bordering the central nervous system (CNS) parenchyma are the choroid (underlying the retina) and the leptomeninges (the inner layers of the meninges enveloping the brain). Although near the neural parenchyma, the choroid and leptomeninges are external to the immune privileged environment of the retina and brain and thus are distinct immune compartments. This study aimed to characterize the transcriptomic signatures of immune cells within the choroid and leptomeninges bordering the healthy adult mouse CNS.

**Methods:**

Eyes and brains were obtained from 7-week-old C57Bl/6J mice. Choroid and leptomeninges were processed for isolation of CD45^+^ immune cells and single cell RNA-sequencing. Additionally, single cell RNA-sequencing was performed on immune cells isolated from choroid obtained from human donor eye tissue. Immunostaining and confocal microscopy of wholemount tissue were used to validate selected immune cell populations in situ.

**Results:**

A total of 3606 cells were sequenced from mouse tissues, including 2125 CD45^+^ cells from choroid and 1481 CD45^+^ cells from leptomeninges. Clustering and differential gene expression analysis revealed heterogeneous subtypes of monocytes/macrophages, dendritic cells, T cells, and B cells. Whereas some clusters were common to both choroid and leptomeninges, others exhibited tissue-specific gene expression profiles and potential functional specializations. Analysis of 6501 CD45+ cells sequenced from human choroid identified similar immune cell populations to mouse choroid.

**Conclusions:**

This study provides a detailed characterization of the molecular signatures of immune cells within the vascular connective tissues bordering the healthy retina and brain, and their potential roles in immune protection.

The central nervous system (CNS; comprising the brain, spinal cord, and neural retina) is a complex organ system that requires specialized support from bordering tissues and spaces. The bordering tissue of the retina is the choroid, a highly vascularized layer underlying the retinal pigment epithelium (RPE); and the ciliary body, which secretes aqueous humour and forms part of the blood-ocular barrier.^1^^,^^2^ Bordering the brain are the meninges, comprising three layers that cover the brain; and the choroid plexus within the ventricles, which secrete cerebrospinal fluid (CSF) and form the blood-CSF barrier.^3^^,^^4^ The pia mater and arachnoid mater (the leptomeninges) are the inner and middle layers of the meninges, respectively. Both the leptomeninges of the brain and choroid of the eyes have vastly different immune properties compared to the underlying neural parenchyma.^2^ As the immunological interface between the periphery and neural environment, the choroid and leptomeninges play important roles in immune surveillance and defense against pathogens and insults.^1^^,^^5^

Beyond their role in immune defense, tissue resident immune cells contribute to homeostatic functions.^6^^,^^7^ Defining the diversity of immune cells within the choroid and meninges is therefore important for advancing our understanding of the immunobiology at the borders of the retina and brain. Unlike the CNS parenchyma, which is populated by microglia, the choroid and meninges contain macrophages, dendritic cells, mast cells, and lymphocytes.^7^^–^^11^ Seminal studies of border-associated macrophages (BAMs) within the mouse meninges, choroid plexus, and perivascular space demonstrated that these cells exhibit region-specific transcriptomic signatures and ontogenies.^8^^,^^9^ However, comparative studies of immune cells within the bordering tissues of the brain and retina are lacking. Because increasing evidence suggests that immune cell identities are shaped by their tissue environment, we hypothesized that the choroid and leptomeninges would contain similar types of immune cells but would be distinguished by tissue-specific gene expression profiles.

Advances in single-cell RNA sequencing (scRNA-seq) technology have enabled more detailed molecular characterization of immune cell subtypes at an unprecedented level of resolution. We therefore used scRNA-seq to profile the immune landscape of the choroid and leptomeninges bordering the healthy mouse retina and brain, and identify tissue-specific transcriptomic signatures.

## Methodology

### Mice, Tissue Collection, and Dissection

Mouse experiments were approved by the QIMR Berghofer Medical Research Institute Animal Research Ethics Committee (approval A18613M); QUT AEC administrative approval 1800001261. All manipulations were performed in accordance with the Association for Research in Vision and Ophthalmology Statement for the Use of Animals in Ophthalmic and Vision Research and the National Health and Medical Research Council Australian Code for the Care and Use of Animals for Scientific Purposes. A notifiable low risk dealing for the use of genetically modified mice in this project was obtained from the QUT University Biosafety Committee (NLRD approval 1800000957).

Adult (7 weeks of age) female C57Bl/6J mice were used for this study. C57Bl/6J mice were purchased from the Animal Resources Centre (Canning Vale, Western Australia). Mice were maintained on a 12:12 hour light cycle with access to food and water ad libitum. For experiments, mice were deeply anesthetized by an intraperitoneal injection of sodium pentobarbital (150 mg/kg). Mice were then perfused immediately through the left ventricle with cold phosphate buffered saline (PBS) to clear the vasculature of blood components including circulating leukocytes. When tissues were being collected for histology and immunostaining, the mice were also transcardially perfused with 4% paraformaldehyde (PFA; Sigma Aldrich P6148) to deliver fixative systemically to the animal.

Brains and eyes were collected from perfused mice and dissected under a stereomicroscope. During mouse brain collection, the calvaria (containing dura mater and likely part of the arachnoid)^12^^,^^13^ was removed, leaving leptomeninges adherent to the surface of the brain. To obtain samples of leptomeninges, the superior surface of the cerebral cortex was trimmed using a flat-edged razor blade, as previously described.^14^ The resulting sample that was processed for scRNA-seq was enriched for pial leptomeninges but also contained a small amount of underlying cortical tissue. Choroids were collected as previously described.^15^

### scRNA-Seq of Immune Cells Within the Choroid and Leptomeninges

Immune cells (CD45^+^ cells) were isolated from the choroid and leptomeninges obtained from 20 C57Bl/6J female mice by fluorescent activated cell sorting (FACS). Freshly dissected choroid and leptomeninges were pooled to obtain a sufficient number of cells. The dissected tissues were transferred to 50 mL tubes containing dissection buffer (1 × HBSS, no calcium, no magnesium, no phenol red; 5 mM glucose; and 15 mM HEPES) and all processing steps were performed on ice. The tissues were passed through a 70 µm nylon cell strainer (Falcon 352350) using the plunger of a 5 mL syringe to obtain single cell suspensions, and then pelleted by centrifugation at 400 *g* for 5 minutes at 4°C. Leptomeninges were subsequently resuspended in 30% (v/v) Percoll (GE Healthcare 17089102) in 1 × PBS and centrifuged at 700 *g* for 10 minutes at 4°C without a brake being applied. The small, myelinated layer formed at the top of the tube (from cortical tissue) was discarded prior to resuspending the cell pellet. The number of viable cells was determined by exclusion of Trypan blue stain as viewed by microscopy, assisted by use of a hemocytometer. Cells were subsequently resuspended in FACS buffer (3 mM EDTA, 0.1% [w/v] bovine serum albumin [BSA], 1 × PBS, and 100 µg/mL DNase I) and stained with rat anti-mouse CD16/CD32 (0.5 µg per million cells; BD Biosciences 553141) for 15 minutes, then centrifuged at 400 *g* for 5 minutes. The pellet was resuspended in FACS buffer containing BV421 rat anti-mouse CD45 antibodies (0.25 µg per million cells; BD Biosciences 563890), incubated for 30 minutes on ice, and then centrifuged and resuspended in FACS buffer. CD45^+^ were isolated from stained cell suspensions using a BD FACS Aria IIIu (100 µm nozzle) at QIMR Berghofer Flow Cytometry Facility. Dead cells were identified and excluded during sorting by staining with propidium iodide (1 µg/mL, BD Biosciences 556463).

Sorted cells were partitioned into single cell droplets using the 10X Genomics Chromium Controller. Subsequently, scRNA-seq libraries were prepared using a Chromium single cell reaction 3′ version 3.1 kit (10X Genomics). Single cell libraries were sequenced on an Illumina NovaSeq S1 instrument, producing paired-end reads.

### scRNA-Seq of Human Choroidal Immune Cells

Human tissue experiments were approved by Metro South Human Research Ethics Committee (HREC/07/QPAH/048); QUT HREC administrative approval 0800000807/ERM Project No. 5297. For the study of human choroidal immune cells, scRNA-seq was performed on cells isolated from human postmortem eyes. Fresh (unfixed) human eye cups from a 45-year-old male donor with no history of eye disease or diabetes was obtained within 17 hours postmortem from the Queensland Eye Bank. The vitreous and retina were removed from the eye cups and choroid-RPE was subsequently dissected from the sclera. Choroid-RPE tissue was transferred into a 12-well plate and subjected to enzymatic digestion with collagenase type II (200 U/mL; Life Technologies 17101-015) in HBSS (calcium, magnesium, no phenol red) for 60 minutes at 37°C, with tissue triturated every 15 minutes. After 60 minutes, choroidal tissue was triturated with a pipette and then passed through a 70 µm cell strainer to generate a single cell suspension. The cell suspension was pelleted by centrifugation at 400 *g* for 5 minutes, resuspended in freezing medium (DMEM/F12 [Gibco 11320033] containing 10% [v/v] DMSO and 10% [w/v] BSA), and transferred to a Cool Cell freezing chamber at –80°C for controlled freezing. The cells then were transferred to liquid nitrogen storage. For scRNA-seq studies, cells were thawed and pre-warmed DMEM/F12 containing 10% (w/v) BSA was added in a dropwise manner. The viability and yield of thawed cells were determined by Trypan blue staining using a hemocytometer.

Human choroidal immune cells were isolated from thawed cell suspensions by FACS. Thawed cell suspensions were incubated with anti-human Fc Block (2.5 µg per million cells; BD Biosciences 564220) for 15 minutes, and then centrifuged at 400 *g* for 5 minutes. The pellet was resuspended in FACS buffer containing BV421 mouse anti-human CD45 antibodies (2.5 µL per million cells; BD Biosciences 563879) and incubated on ice for 30 minutes. The cells were centrifuged at 400 *g* for 5 minutes and then resuspended in FACS buffer for sorting. Live (propidium iodide negative) immune cells (CD45^+^) were sorted using a BD FACS Aria IIIu with a 100 µm nozzle.

The isolated cells were partitioned into single cell droplets using the 10X Genomics Chromium Controller and scRNA-seq library preparation was completed using the Chromium 3′ version 3.1 single cell reaction kit (10X Genomics). The single cell library was sequenced on an Illumina NovaSeq S1 instrument, producing paired end reads.

### scRNA-Seq Data Analysis: Processing, QC Metrics, Removing Non-Target Cells, and Clustering

Sequences were initially processed using 10X Genomics Cell Ranger version 7.0.0 software^16^ on a Linux HPC (high performance computing) environment. The Cell Ranger workflow consisted of two primary steps: first “cellranger mkfastq” was used to demultiplex raw base call data files and convert these to fastq format files; second, “cellranger count” was used to align sequences to the mouse reference genome, and then quantify the number of aligned sequences per genomic feature (i.e. defined gene regions), producing a count table that formed the basis of further downstream analysis (dimensionality reduction, clustering, and visualization). Additionally, “cellranger aggr” was run to aggregate the output from multiple cellranger count runs (samples). Counts were re-quantified based on relative library size (normalization) and gene expression was re-calculated.

The count table generated by Cell Ranger was imported into R software version 4.0.5 (R Core Team, 2022)^17^ using the “Read10X” function from the Seurat package.^18^ This was combined with the cell IDs (barcodes.tsv.gz) the gene IDs (features.tsv.gz) into a single Seurat database object using the “CreateSeuratObject” function. Raw count data were transformed via dimensionality reduction so Principal Component Analysis (PCA), t distributed stochastic neighbor embedding (t-SNE) and Uniform Manifold Approximation Projection (UMAP) plots could be generated. This involved: (1) normalization of data by log transformation, (2) identification of genes that exhibited high cell-to-cell variation, (3) scaling the data so that highly expressed genes did not dominate the visual representation of expression, (4) performing the linear dimensional reduction that converts expression to dimensions, and (5) plotting the first two dimensions in a PCA, t-SNE, or UMAP plot. Each of these steps was achieved using Seurat functions.

Prior to clustering, filtration was applied to identify and remove outlier cells with aberrantly high (>4000) or low (<200) gene counts. Optimal clustering was guided by the Clustree package,^19^ which facilitated identification of optimal resolution scores to prevent over- or under-clustering for each dataset. Once quality filtration was completed, the cells were clustered by gene expression using Seurat's “FindClusters” function and clustering was visualized with t-SNE, dot plots, and heat maps.

### scRNA-Seq Data Analysis: Identifying Differentially Expressed Genes, Cell Subtypes, and Functional Enrichment

Differentially expressed genes (DEGs) were identified using Seurat's “FindMarkers” function. Significantly differentially expressed genes were defined as having an adjusted *P* < 0.05 and log fold change (FC) > 0.5. Initially, clusters were identified as immune cell types based on their expression of common signature genes (Supplementary Table S1).

DEGs were then examined for functional enrichment in Gene Ontology (GO) terms using the package clusterProfiler.^20^ Annotation of primary gene IDs (gene symbol) to other gene identifiers (such as Entrez gene ID and Ensembl gene ID) was completed using the AnnotationHub package.^21^

### Spatial Validation of scRNA-Seq Data Using Immunohistochemistry and Confocal Microscopy

Immunohistochemistry was performed on fixed wholemount choroid and leptomeninges obtained from C57Bl/6J mice. CD19 polyclonal antibodies (1:200 dilution; Invitrogen PA5-114970) and CD3 monoclonal antibodies (1:400 dilution; Invitrogen 14-0032-82) were used to label B cells and T cells, respectively. To achieve this, wholemount choroid and leptomeninges were permeabilized in 0.5% (v/v) Triton X-100 (Sigma Aldrich X100-100ML) in PBS at room temperature for 1 hour. The tissues were then blocked in 3.0% (w/v) BSA (Sigma Aldrich A7906-500G) and 0.3% (v/v) Triton X-100 in PBS for 1 hour at room temperature. Tissues were then incubated with primary antibodies diluted in blocking solution overnight at 4°C. Tissues were washed 3 times (each for 10 minutes) in PBS, and then incubated with fluorophore-labeled secondary antibodies Goat anti-rabbit Alexa Fluor Plus 488 (1:400 dilution; Life Technologies A32731) for CD19, and Goat anti-rat Alexa Fluor 647 (1:400 dilution; Life Technologies A21247) for CD3, along with Hoechst (1:1000 dilution; Life Technologies 62249) for 2 hours at room temperature. Tissues were washed three times (each 10 minutes) in PBS, mounted on microscope slides (SuperFrost Plus White 1 mm slide, Thermo Fisher Scientific MENSF41296SP) and coverslipped (Coverglass, rectangular, 22 × 40 mm, 1.5 thickness, ProSciTech G425-2240). Samples were imaged using an inverted SP5 five-channel confocal microscope (Leica Microsystems). Z stacks were captured every 1 µm, with a line averaging value of 3 applied. Maximum intensity projection images were created using FIJI.^22^

### Data Availability

The scRNA-seq data generated in this study have been deposited in NCBI’s Gene Expression Omnibus^23^ and are accessible through GEO Series accession number GSE253419 (https://www.ncbi.nlm.nih.gov/geo/query/acc.cgi?acc=GSE253419).

## Results

### Identification of Immune Cell Types in Mouse Choroid and Leptomeninges

**Figure 1.**
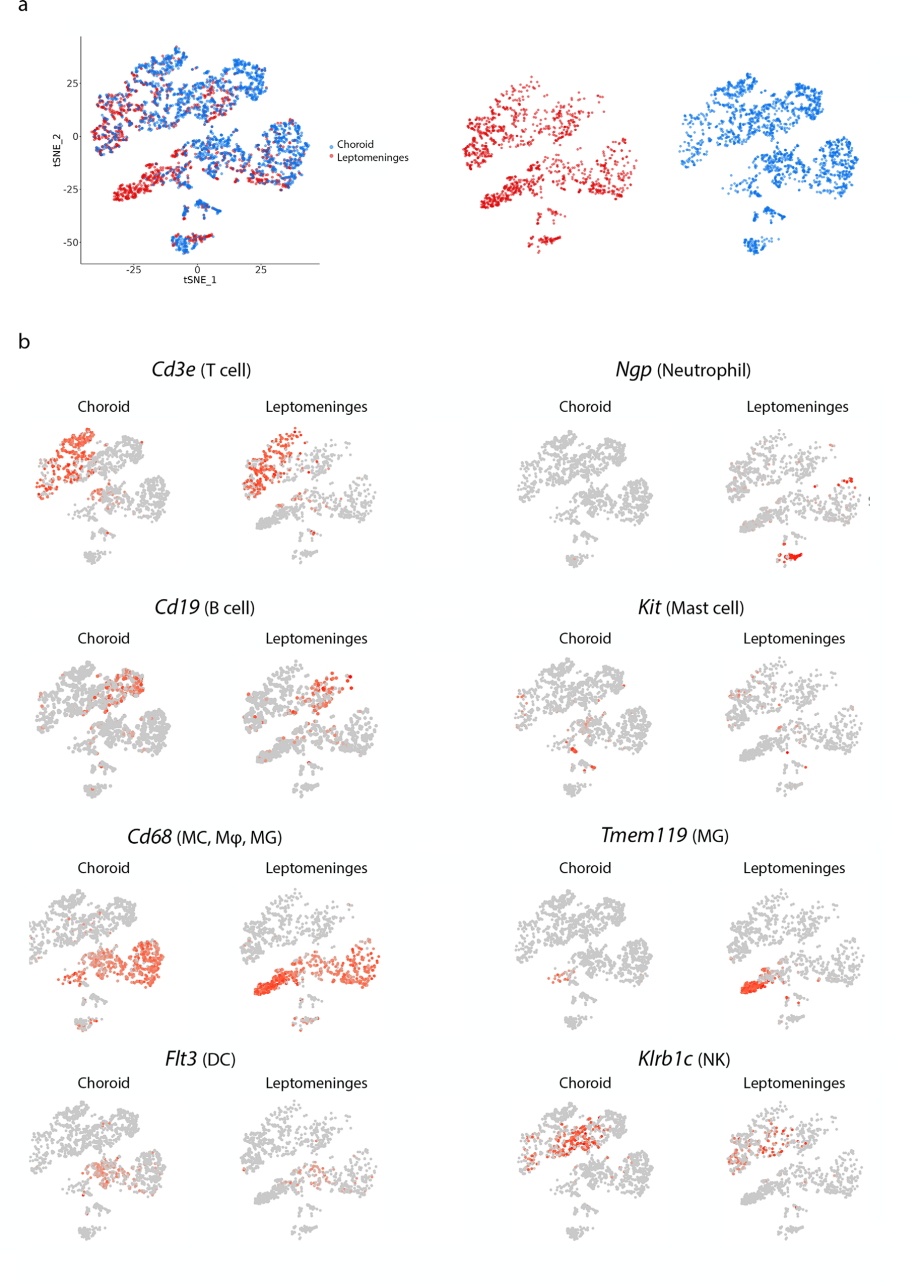
**Comparison of immune cell types in mouse choroid and leptomeninges.** (**a**) t-SNE plot of 3606 immune cells (CD45^+^) sorted from the choroid and leptomeninges of C57BL/6J mice (tissue pooled from 20 mice). Cells are colored according to their origin (choroid = *blue*, and leptomeninges = *red*). (**b**) t-SNE maps showing the frequency of cells expressing genes used to identify immune cell clusters (*grey dots* indicate cells not expressing the gene; and the *red dots* indicate cells expressing the gene). MC, monocyte; Mφ, macrophages; DCs, dendritic cells; NK, natural killer cells; MG, microglia.

To characterize immune cell populations in the bordering tissues of the mouse brain and retina, we sorted CD45^+^ immune cells from choroid and leptomeninges obtained from 7-week-old female C57Bl/6J mice and performed scRNA-seq. Unsupervised clustering and t-SNE projections were performed on aggregated data from both tissues (3606 total cells; Fig. 1a), and clusters were identified based upon their expression of known marker genes (see Supplementary Table S1). Significant overlap was observed between immune cell types isolated from choroid and leptomeninges; however, some tissue-specific differences were detected (Fig. 1b). Both tissues contained populations of monocytes/macrophages (MC/Mφ), dendritic cells (DCs), T cells, B cells, and natural killer (NK) cells. Neutrophils were only detected within leptomeninges. Small numbers of mast cells were detected in both tissues (mostly in the choroid); however, these did not form distinct clusters within t-SNE plots. Microglia (MG) from the underlying neural tissue were also detected, particularly in leptomeninges, which cannot be completely peeled away from the surface of the mouse brain.

### Characterization of Immune Cell Clusters in the Mouse Choroid and Their Unique Transcriptomic Signatures

**Figure 2.**
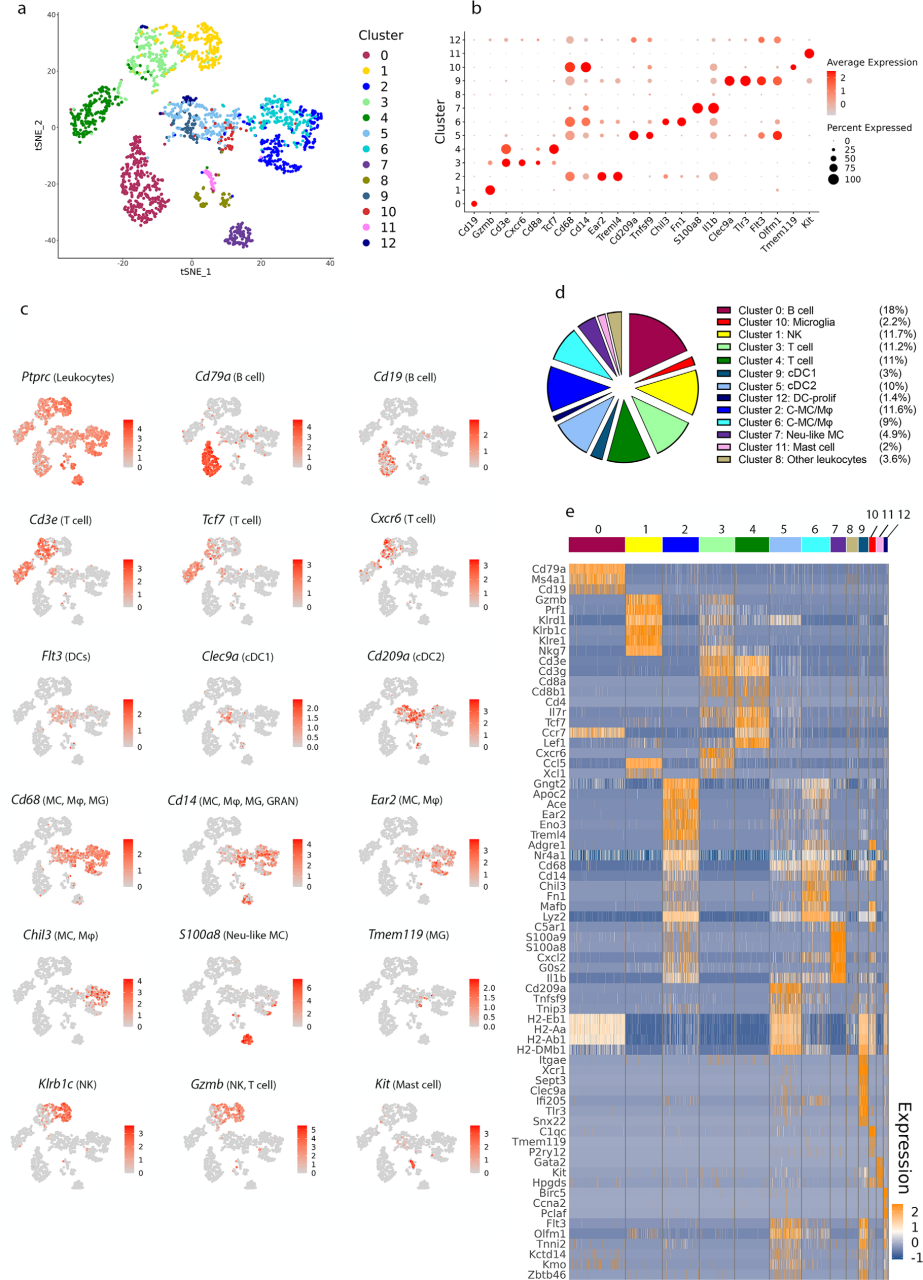
**Identifying immune cell types in the mouse choroid based on differentially expressed genes.** (**a**) t-SNE plot of 2125 immune cells (CD45^+^) sorted from choroid tissue pooled from 20 C57Bl/6J mice, showing the thirteen immune cell types that were identified via unsupervised clustering. (**b**) Dot plots showing the expression of cell type-specific genes, with the dot size representing the percentage of cells expressing the gene and the color representing its average expression within a cluster. (**c**) t-SNE maps showing the expression of key genes for the immune cell populations that identified in **b**. *Grey* indicates low expression; *red* indicates high expression, and range of log_2_ normalized counts are shown on the right of each plot. (**d**) Pie charts showing the proportions of different immune cell types within the homeostatic mouse choroid. Clusters were identified based on expression of specific genes (the pie chart legend is ordered according to cell type rather than by numerical order of clusters). The frequency of each cluster is shown as the percentage of the total immune cell population to the right of each cluster name. Note that the cluster identified as “other leukocytes” expressed the CD45 gene (*Ptprc*), but none of the other examined lineage markers. (**e**) Heatmap of normalized expression for selected differentially expressed genes in each cluster. Range of log_2_ normalized counts are shown at right hand of heatmap. *Blue* indicates low expression; and *orange* indicates high expression. C-MC/Mφ, choroidal monocyte/macrophage; DCs, dendritic cells; NK, natural killer cells; MG, microglia; GRAN, granulocytes; Neu-like MC, neutrophil-like monocyte.

To generate a transcriptomic profile of leukocytes within the homeostatic adult choroid, cells from choroid tissue were re-analyzed in a single dataset separate from leptomeninges. Unsupervised clustering and t-SNE projections were performed on 2125 CD45^+^ cells (229,696 mean reads per cell; Fig. 2a), and clusters were identified based upon the expression of cell-specific genes (Figs. 2b–e). Thirteen immune cell clusters were resolved, and these clusters were identified based on their expression of cell-specific genes, including various subtypes of B cells (*Cd79a*^+^ and *Cd19*^+^), T cells (*Cd3e*^+^), DCs (*Flt3*^+^), MC/Mφ/ granulocytes (GRAN; *CD68*^+^ and *Cd14*^+^), NK cells (*Klrb1c*^+^ and *Gzmb*^+^), and mast cells (*Kit*^+^; see Figs. 2b–e). Myeloid cells and T cells were the most abundant immune cell types within the choroid (see Fig. 2d) and were resolved into several clusters based on DEGs, as described below.

Three DC clusters were observed in the choroid. Each of these clusters expressed *Flt3*, which is required for DC development and homeostasis,^24^ and the DC-specific transcription factor *Zbtb46.*^25^ The gene expression profiles of these clusters were consistent with conventional type 1 DCs (cDC1, cluster 9), cDC2 (cluster 5), and proliferative DCs (DC-prolif, cluster 12). Both cDC1 and cDC2 clusters expressed antigen processing and presentation related genes, such as *H2-Eb1*, *H2-Aa*, *H2-Ab1*, and *H2-DMb1*. cDC1s were distinguished by 302 DEGs, including *Itgae*, *Xcr1*, *Sept3*, *Clec9a*, *Pianp*, *Ffar4*, *Plpp1*, *Ifi205*, *Tlr3*, and *Snx22*. The cDC2 cluster was the predominant DC population within the choroid and cells within this cluster were enriched for *Il3ra*, *Cd209a*, *Tnfsf9*, and *Tnip3*, and had 252 DEGs. DC-prolif (cluster 12) expressed genes, such as *Birc5*, *Ccna2*,^26^ and *Pclaf*^27^ that promote proliferation, in addition to the common DC markers. The functional implications of proliferative gene expression by this cluster are unclear, as it would be unlikely that DCs in the healthy choroid undergo active proliferation.

Two distinct choroidal MC/Mφ clusters (clusters 2 and 6) and one neutrophil-like monocyte (Neu-like MC) cluster (cluster 7) with unique transcriptomic signatures were resolved in the choroid. Cluster 2 was the predominant MC/Mφ cluster and was distinguished by 689 DEGs including *Gngt2*, *Apoc2*, *Ace*, *Eno3*, and *Treml4*. Choroidal MC/Mφ cluster 6, characterized by 577 DEGs, expressed genes *Chil3*, *Fn1*, *Mafb*, and *Lyz2*. Cluster 7 was unique compared to the other choroidal MC/Mφs due to its low expression of *Cd68*, yet enrichment of *Cd14*, *S100a9*, *S100a8*, and *Il1b*. The gene expression of this cluster resembled neutrophil-like monocytes (Neu-like MC), which were recently identified in an anti-glomerular basement membrane crescentic glomerulonephritis mouse model^28^^,^^29^; however, further work is needed to determine the identity of this cluster.

Two T cell clusters (cluster 3 and cluster 4; characterized by expression of *Cd3e*, *Cd3g*, and *Cd3d*) were identified in the choroid. Cluster 3 expressed genes consistent with CD8^+^ T cells (*Cd8a*, *Cd8b1*, *Nkg7*, and *Gzmb*) and was also characterized by DEGs, including *Cxcr6* and *Ccl5*. Cluster 4 potentially contained a mixture of T cell subtypes, as this cluster expressed *Cd8a*, *Cd8b1*, and *Cd4*. This cluster was also enriched for *Il7r* and *Ccr7*, which are expressed by CD8^+^ and CD4^+^ effector memory T cells, as well as *Lef1* and *Tcf7*, which are expressed by naïve T cells. *Tcf7* is a transcription factor that plays an important role during T cell development and differentiation.^30^^,^^31^ It is possible that this putative mixture of T cell subtypes could be resolved into individual clusters with increased numbers of sequenced cells.

### Characterization of Immune Cell Clusters in Mouse Leptomeninges and Their Unique Transcriptomic Signatures

**Figure 3.**
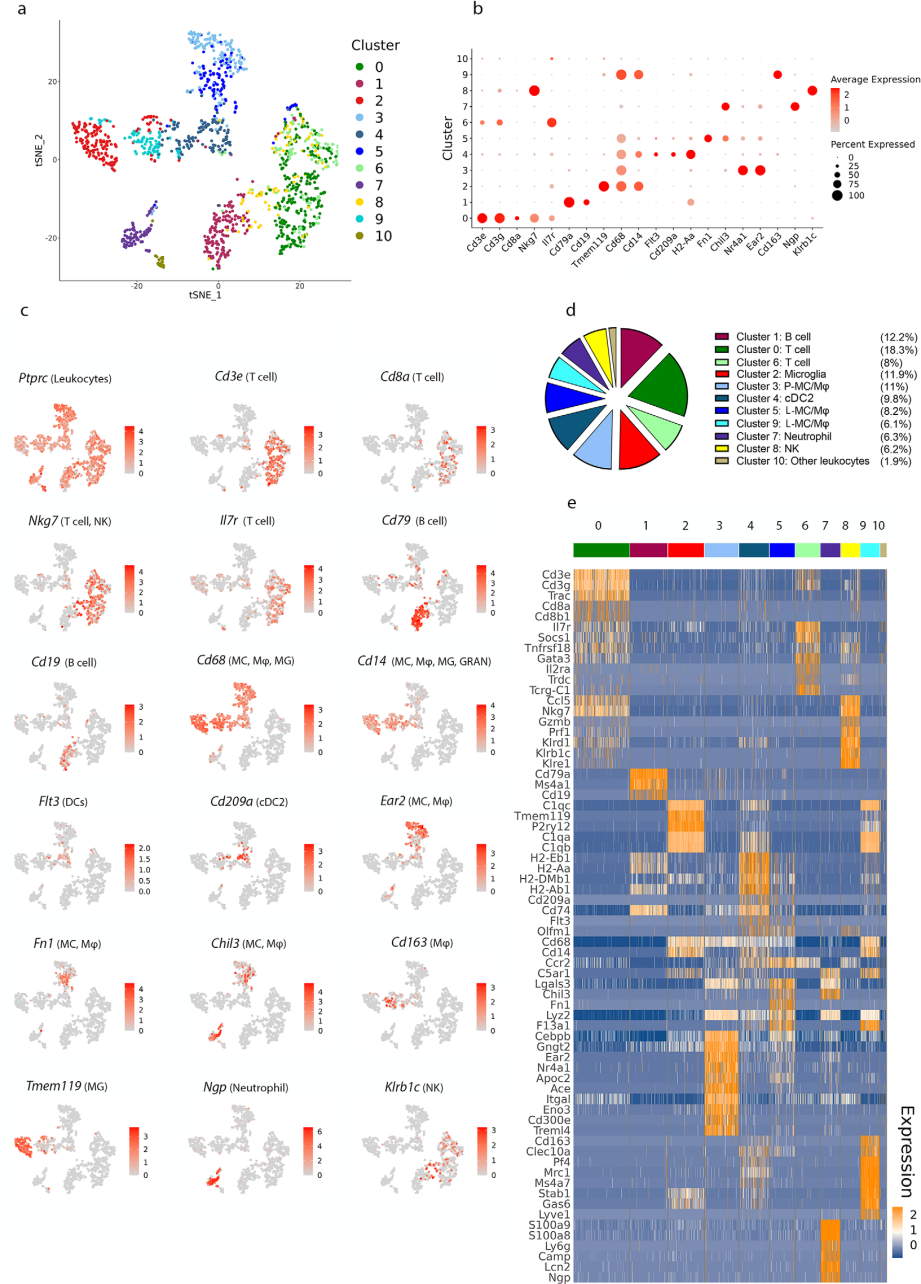
**Identifying immune cell types in mouse leptomeninges based on differentially expressed genes.** (**a**) The t-SNE plot of 1481 immune cells (CD45^+^) sorted from leptomeninges pooled from 20 C57Bl/6J mice, showing the eleven immune cell clusters that were identified via unsupervised clustering. (**b**) Dot plots demonstrating the expression of cell type-specific genes, with the dot size representing the percentage of cells expressing the gene and the color representing its average expression within a cluster. (**c**) The t-SNE maps showing the expression of key marker genes for the immune cell populations that were identified in **b**. Grey, low expression; red, high expression, and range of log_2_ normalized counts are shown on the right of each plot. (**d**) Pie chart showing the proportions of different immune cell clusters within leptomeninges. Clusters were identified based on expression of specific genes (the pie chart legend is ordered according to cell type rather than by numerical order of clusters). The frequency of each cluster is shown as percentage of the total immune cell population to the right of each cluster name. Note that the cluster identified as “other leukocytes” expressed the CD45 gene (*Ptprc*), but none of the other examined lineage markers. (**e**) Heatmap of normalized expression for selected genes in each cluster. Range of log_2_ normalized counts are shown at right hand of heatmap (*blue* indicates low expression and *orange* indicates high expression). DCs, dendritic cells; GRAN, granulocytes; L-MC/Mφ, leptomeningeal monocyte/macrophage; MG, microglia; NK, natural killer cells.

Using the same approach as described above for the choroid, a transcriptomic profile of immune cells within the homeostatic mouse leptomeninges was generated by performing unsupervised clustering and t-SNE projections on 1481 CD45^+^ cells (324,070 mean reads per cell; Fig. 3a). Eleven immune cell clusters were detected including T cells (*Cd3e*^+^), B cells (*Cd79a*^+^ and *Cd19*^+^), MC/Mφ (*Cd14*^+^ and *Cd68*^+^), DCs (*Flt3*^+^), neutrophils (*Ngp*^+^), and NK cells (*Prf1*^+^ and *Klrb1c*^+^; Figs. 3b–e). Similar to the mouse choroid, several clusters of myeloid cells and T cells were identified.

Clusters 3, 4, 5, and 9 were identified as myeloid cells, which were the most abundant immune cells detected within leptomeninges. Cluster 4 expressed *Flt3*, and had a transcriptional profile consistent with cDC2s. This cluster was distinguished by 133 DEGs, including *Cd209a*, *Cd74*, and antigen processing and presentation genes *H2-Eb1*, *H2-Aa*, *H2-Ab1*, and *H2-DMb1*. Three distinct leptomeningeal MC/Mφ clusters were identified (clusters 3, 5, and 9); each of these clusters expressed the common MC/Mφ gene *Cd68* and had low expression of *Flt3*. Leptomeningeal MC/Mφ cluster 3 expressed 678 DEGs, including *Ear2* and *Nr4a1*, and other genes including *Gngt2*, *Apoc2*, *Ace*, *Itgal*, *Eno3*, *Cd300e*, and *Treml4*. Leptomeningeal MC/Mφ cluster 5 was characterized by 404 DEGs, including *Chil3* and *Fn1*. Finally, leptomeningeal MC/Mφ cluster 9 was distinguished from other MC/Mφs by expression of complement C1q genes *C1qa*, *C1qb*, and *C1qc*. This cluster featured 883 DEGs, which also included *Cd163*, *Clec10a*, *Pf4*, *Mrc1*, *Ms4a7*, *Stab1*, *Gas6*, and *Lyve1*.

Two leptomeningeal clusters (cluster 0 and cluster 6) were identified as T cells based on their expression of *Cd3e*, *Cd3g*, and *Cd3d*. Cluster 0 expressed genes consistent with CD8^+^ T cells (*Cd8a*, *Cd8b1*, and *Nkg7*), and effector memory T cells (*Il7r*), and was enriched for *Ccl5* expression. In contrast, cluster 6 T cells were characterized by expression of *Socs1*, *Tnfrsf18*, and *Il2ra* (enriched in activated CD4^+^ T cells and CD4^+^ regulatory T cells), and *TcrgC1* (the gamma T cell receptor gene that is expressed by gamma-delta T cells). These findings suggest that the two identified leptomeningeal T cell clusters contain various smaller subtypes of T cells.

### Dendritic Cells in the Bordering Tissues of the Mouse Retina and Brain Exhibit Tissue-Specific Transcriptional Signatures

**Figure 4.**
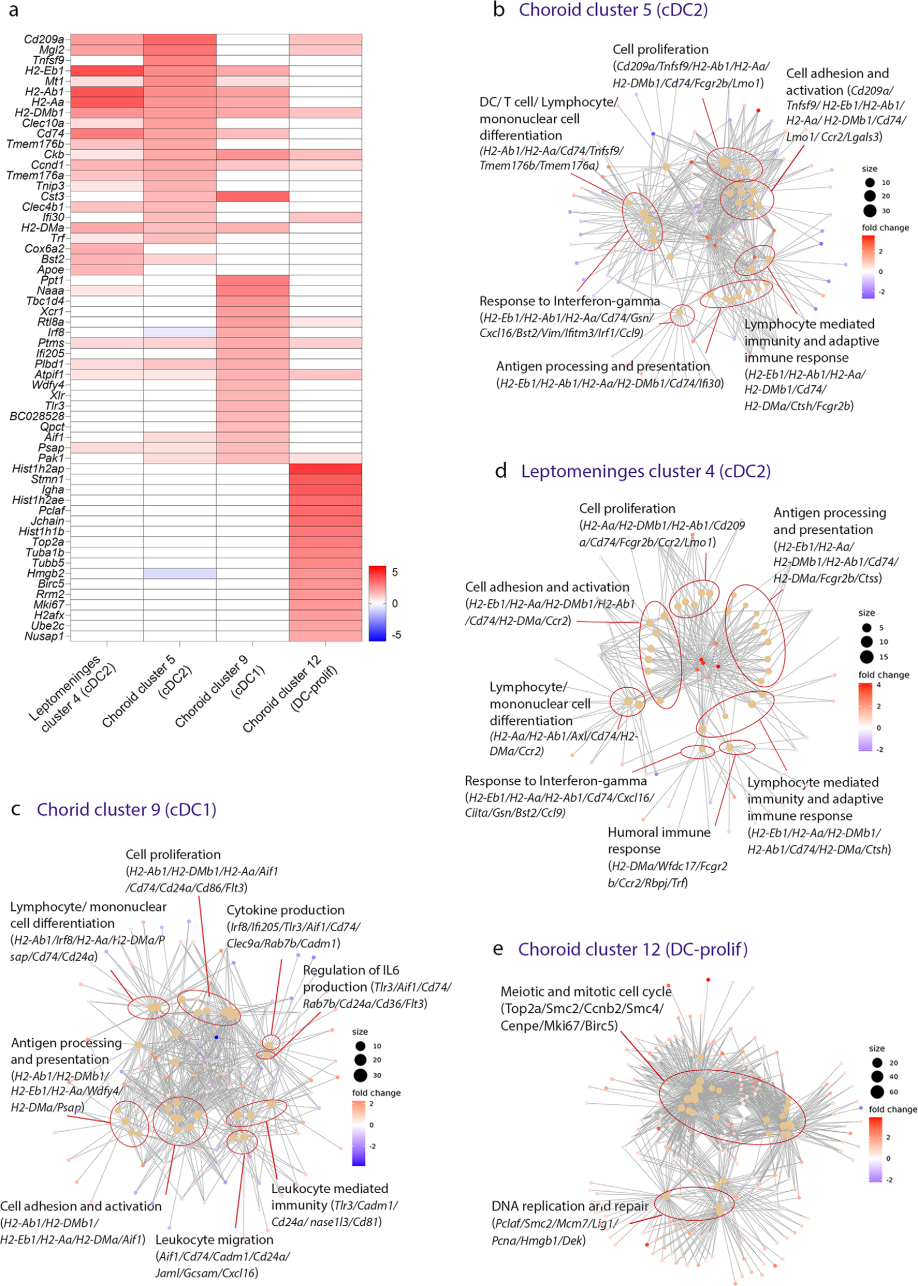
**Comparison of over** **-** **represented GO terms of DC clusters within mouse choroid and leptomeninges.** (**a**) Heat map of normalized gene expression showing the top upregulated DEGs (log_2_ fold change of > 1.5) in leptomeningeal DC2 (cluster 4), choroidal DC2 (cluster 5), and choroidal DC1 (cluster 9) clusters, and top upregulated DEGs (log_2_ fold change of > 2) in DC-prolif (cluster 12), compared to other clusters in leptomeninges and choroid respectively. Range of log_2_ normalized counts are shown to the right of heatmap. (**b**–**e**) Gene ontology network of top 30 GO terms based on genes that are differentially expressed between each DC cluster and other subsets in either leptomeninges or choroid. Each node (*filled dots*) represents a gene ontology and the size of each node reflects the p values of the terms, with the more significant terms being larger. The main themes within the data are categorized into groups (*red circles*). A selection of key genes associated with each cluster of gene ontologies is displayed.

Our analysis revealed that the mouse choroid contained three DC clusters, whereas leptomeninges contained only one DC cluster. To further characterize DC heterogeneity between these tissues, we compared DEGs between DC clusters. The leptomeningeal cDC2 cluster (cluster 4) shared a similar transcriptional signature to the choroidal cDC2 cluster (cluster 5). However, some differences were identified between these two clusters; for example, *Tnfsf9*, *Mt1*, *Tnip3*, and *Ifi30* were highly expressed in choroidal cDC2s, whereas *Cox6a2*, *Bst2*, and *Apoe* was enriched in the leptomeningeal cDC2 cluster (Fig. 4a). GO analysis demonstrated that MHC class II genes, *Cd209a*, *Cd74*, *Fcgr2b*, *Lmo1*, and *Ccr2* were involved in the top 30 over-represented GO terms for both the leptomeningeal cDC2 cluster and choroidal cDC2 cluster (Figs. 4b, 4d). Cell proliferation, cell adhesion and activation, lymphocyte mediated immunity, antigen processing and presentation, cell differentiation, and response to interferon gamma were functions common to leptomeningeal cDC2 and choroidal cDC2 clusters. Genes involved in the top 30 over-represented GO terms for the choroidal cDC1 cluster included MHC class II related genes, *Aif1*, *Cd74*, *Cd24a*, *Cd86*, and *Tlr3*; and regulation of IL-6 production was a top 30 GO term that was unique to this cluster (Fig. 4c). In contrast, the choroidal DC-prolif cluster (cluster 12) shared few genes with the other characterized DC clusters and was enriched for GO terms related to meiotic and mitotic cell cycle and DNA replication and repair (Fig. 4e).

### Mouse Choroid and Leptomeninges Contain Diverse Monocyte/Macrophage Populations With Potential Functional Differences

**Figure 5.**
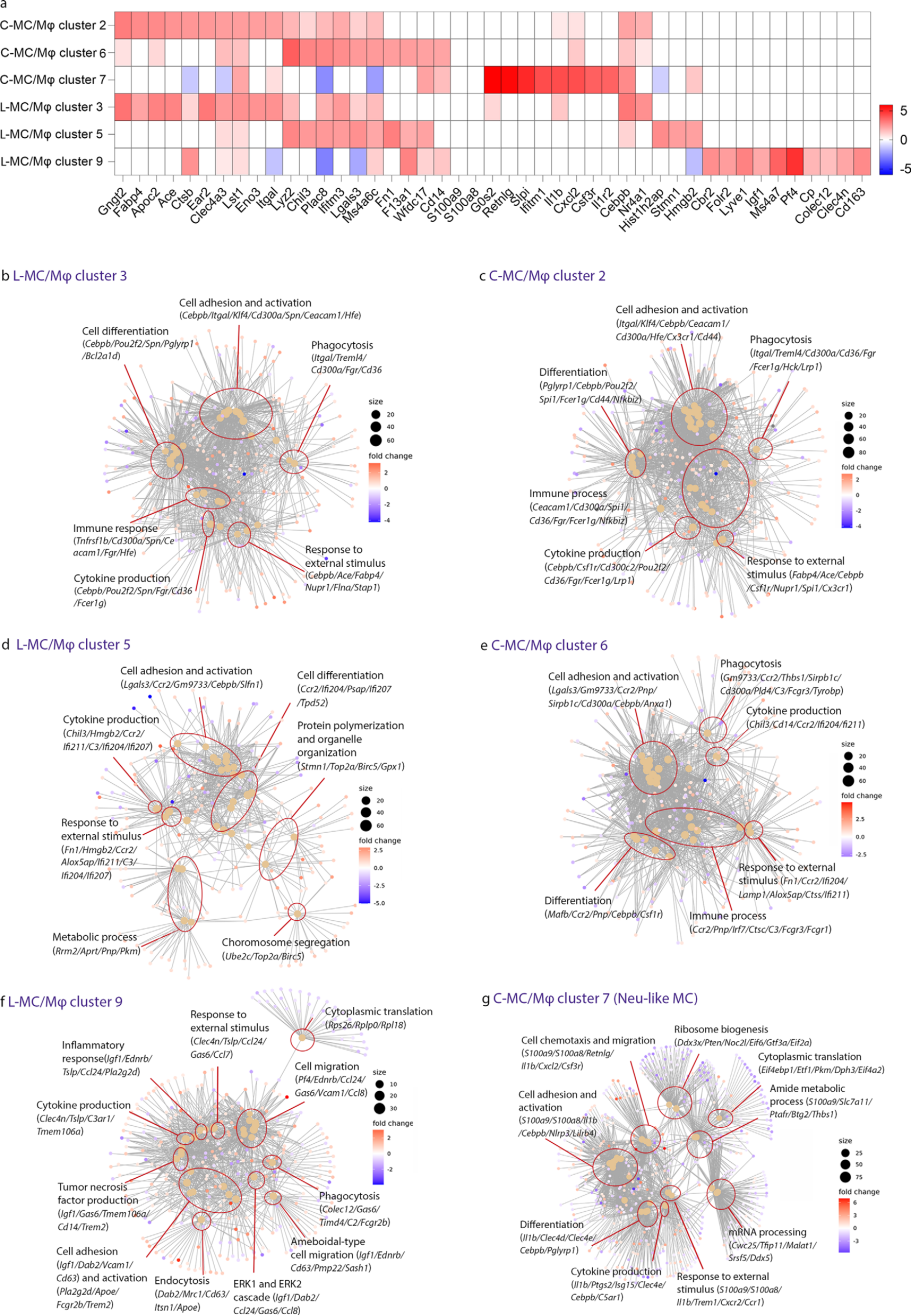
**Comparison of overrepresented GO terms of MC/Mφ clusters within mouse choroid and leptomeninges.** (**a**) Heat map of normalized expression showing the 10-top upregulated DEGs in leptomeninges (L-MC/Mφ clusters 3, 5, and 9) and choroid (C-MC/Mφ clusters 2, 6, and 7), compared to other clusters in the leptomeninges and choroid respectively. Range of log_2_ normalized counts are shown to the right of heatmap. (**b–g**) Gene ontology networks of the top 30 GO terms based on genes that are differentially expressed by each leptomeningeal and choroidal MC/Mφ cluster compared to other clusters within the leptomeninges or choroid, respectively. Each node (*filled in dots*) represents a gene ontology, and the size of the GO terms reflects the p values of the terms, with the more significant terms being larger. The main themes within the data are categorized into groups (*red circles*). A selection of key genes associated with each cluster of gene ontologies is displayed. C-MC/Mφ, choroidal monocyte/macrophage; L-MC/Mφ, leptomeningeal monocyte/macrophage.

To further delineate the heterogeneity of MC/Mφ across these tissues, we conducted a comparison of the top DEGs among MC/Mφ clusters. Striking similarities were observed between choroidal MC/Mφ cluster 2 and leptomeningeal MC/Mφ cluster 3; and between choroidal MC/Mφ cluster 6 and leptomeningeal MC/Mφ cluster 5. In contrast, the DEGs defining choroidal MC/Mφ cluster 7 and leptomeningeal MC/Mφ cluster 9 were unique to their respective tissues (Fig. 5a).

Further comparison of the top over-represented GO terms revealed common functions between the DEGs of several of the MC/Mφ clusters, including phagocytosis, cell adhesion and activation, cytokine production, response to external stimulus, immune processes, and cell differentiation (Figs. 5b–g). Leptomeningeal MC/Mφ cluster 3 and choroidal MC/Mφ cluster 2 showed a high degree of similarity, with the genes *Itgal*, *Klf4*, *Treml4*, *Cd300a*, *Cd36*, *Ace*, and *Fabp4* involved in the top 20 over-represented GO terms for both clusters (see Figs. 5b, 5c). Leptomeningeal MC/Mφ cluster 5 and choroidal MC/Mφ cluster 6 also displayed similarities, and the top genes involved in the over-represented GO terms were *Lgals3*, *Gm9733*, *Ccr2*, *Chil3*, *C3*, *Fn1*, *Ifi211*, and *Ifi204* (see Figs. 5d, 5e). However, leptomeningeal MC/Mφ cluster 5 uniquely showed an over-representation of the GO terms metabolic process, chromosome segregation, and protein polymerization and organelle organization, which suggested an enhanced mitotic state within this cluster (see Fig. 5d).

The unique leptomeningeal MC/Mφ cluster (cluster 9) featured *Pf4*, *Dab2*, *Mrc1*, *Gas6*, *Cd63*, *Fn1*, *Ccl24*, and *Clec4n* as the top genes involved in the over-represented GO terms (see Fig. 5f). Unlike other MC/Mφ clusters within leptomeninges and choroid, this cluster featured tumor necrosis factor production, endocytosis, amoeboid migration, and ERK1/ERK2 cascade among the top over-represented GO terms. Choroidal MC/Mφ cluster 7, putatively identified as Neu-like MC, showed enrichment of GO terms related to cell chemotaxis and migration, cytokine production, response to external stimulus, mRNA processing, ribosome biogenesis, cytoplasmic translation, and metabolic processes. *S100a9*, *S100a8*, *Il1b*, *C5ar*, *Cxcl2*, *Cxcr2*, and *Csf3r* were the top genes related to these over-represented pathways (see Fig. 5g).

### Heterogeneous B and T Cell Gene Expression Profiles Within Mouse Choroid and Leptomeninges

**Figure 6.**
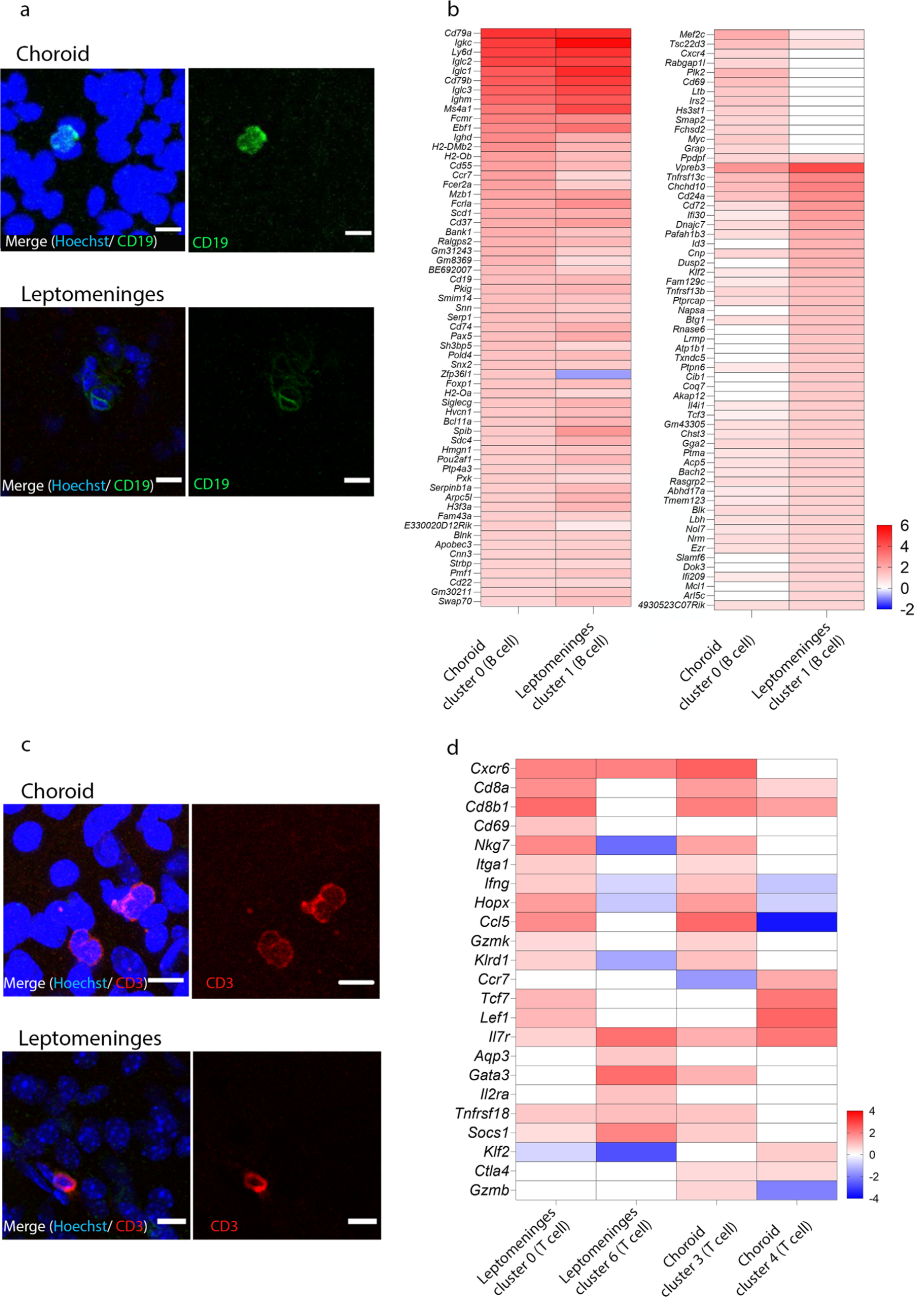
Comparison of lymphocyte cluster DEGs within mouse choroid and leptomeninges. (**a**) Identification of B cells within the healthy mouse (*n* = 1) choroid and leptomeninges using fluorescent immunohistochemistry and confocal microscopy. Immunostaining panels display expression of CD19 (*green*), and blue nuclear staining (Hoechst). Scale bars represent 10 µm. (**b**) Heatmap of normalized expression for top upregulated DEGs (log_2_ fold change of > 1.0) in the choroid B cell (cluster 0) compared to other clusters in the choroid; and leptomeningeal B cell (cluster 1) compared to other clusters in the leptomeninges. Range of log_2_ normalized counts are shown to the right of heatmap. (**c,** **d**) Identification of T cells within the healthy mouse choroid and leptomeninges using fluorescent immunohistochemistry and confocal microscopy. Immunostaining panels display expression of CD3 (*red*), and blue nuclear staining (Hoechst). Scale bars represent 10 µm. (**d**) Heatmap of normalized expression of T cell genes in leptomeninges (cluster 0 and 6) compared to other clusters in the leptomeninges; and in the choroid (cluster 3 and 4) compared to other clusters in the choroid. Range of log_2_ normalized counts are shown to the right of heatmap.

B cells constituted a sizeable proportion of immune cells detected within the healthy mouse choroid and leptomeninges. Although mice were perfused to clear the vasculature of blood components, including circulating leukocytes, prior to tissue collection, complete removal of peripheral components cannot be guaranteed.^32^ Hence, there remains a possibility that a small portion of lymphocytes detected in this study may represent contaminating peripheral immune cells. Regardless, immunostaining and confocal microscopy demonstrated the presence of CD19^+^ B cells in situ within the homeostatic mouse choroid and leptomeninges (Fig. 6a).

The choroidal B cell cluster (cluster 0) was distinguished by 819 DEGs compared to other choroidal clusters, whereas the leptomeningeal B cell cluster (cluster 1) had 771 DEGs relative to other leptomeningeal clusters. The top upregulated DEGs (log_2_ fold change of > 1.0) were compared between leptomeninges and choroidal B cell clusters, and it was found that pan-B cell markers including *Cd79a*, *Cd79b*, and *Cd19* and other B cell markers, such as *Igkc*, *Iglc2*, *Iglc2*, *Iglc3*, and *Ighm* were upregulated in B cell clusters in both tissues. Mature B cell markers such as *Ms4a1*, *Fcer2a*, and *Bank1* were also upregulated in both B cell clusters. However, there were some tissue-specific DEGs that were upregulated in choroidal and leptomeningeal B cell clusters, respectively. For example, *Cxcr4*, *Rabgap1l*, *Plk2*, *Cd69*, *Ltb*, *Irs2*, *Hs3st1*, *Smap2*, *Fchsd2*, *Myc*, and *Grap* were exclusively upregulated in the choroidal B cell cluster. Whereas *Id3*, *Dusp2*, *Napsa*, *Rnase6*, *Lrmp*, *Atp1b1*, *Txndc5*, *Cib1*, *Coq7*, *Akap12*, *Slamf6*, *Dok3*, *Mcl1*, and *Arl5c* were DEGs unique to the leptomeningeal B cell cluster (Fig. 6b). These DEGs between choroidal and leptomeningeal B cell clusters are involved in GO terms including the cellular process, localization, response to stimulus, signaling, developmental process, metabolic process, and immune system process.

Unsupervised clustering of scRNA-seq data revealed that the mouse choroid and leptomeninges contained various T cell clusters (see Figs. 2, 3). Similar to B cells, immunostaining and confocal microscopy confirmed that the naïve mouse choroid and leptomeninges contained CD3^+^ T cells within the tissue (Fig. 6c). To characterize T cell heterogeneity between these tissues, we compared the top upregulated DEGs between leptomeningeal T cell clusters (clusters 0 and 6) and choroidal T cell clusters (clusters 3 and 4; Fig. 6d). Leptomeningeal T cell cluster 0 shared a similar DEGs to the choroidal T cell cluster 3, with *Cd8a*, *Cd8b1*, *Nkg7*, *Il7r*, *Cxcr6*, *Itga1*, *Ifng*, *Hopx*, *Gzmk*, *Klrd1*, and *Ccl5* among the top upregulated DEGs for both clusters. However, some tissue-specific differences were observed, for example, *Cd69*, *Tcf7*, and *Lef1* were upregulated by leptomeningeal T cell cluster 0 (but not choroidal T cell cluster 3), whereas *Ctla4* and *Gzmb* were enriched in the choroidal T cell cluster 3 (but not the leptomeningeal T cell cluster 0). Interestingly, leptomeningeal T cell cluster 6 had a distinct transcriptional profile from leptomeningeal cluster 0 and both choroidal T cells clusters, and was enriched for *Aqp3*, *Socs1*, *Il2ra*, and *Gata3*.

To address the possibility that the identified T cell clusters could represent contaminating peripheral blood T cell subsets, we compared the DEGs for each of the T cell clusters identified in the choroid and leptomeninges with defining genes of T cell subsets in mouse peripheral blood.^33^ We did not observe a significant overlap of genes (Supplementary Table S2), indicating that the identified choroid and leptomeningeal T cell clusters in our study do not share defining gene signatures of T cells within peripheral blood.

### Transcriptomic Profile of Leukocytes Within the Human Choroid

**Figure 7.**
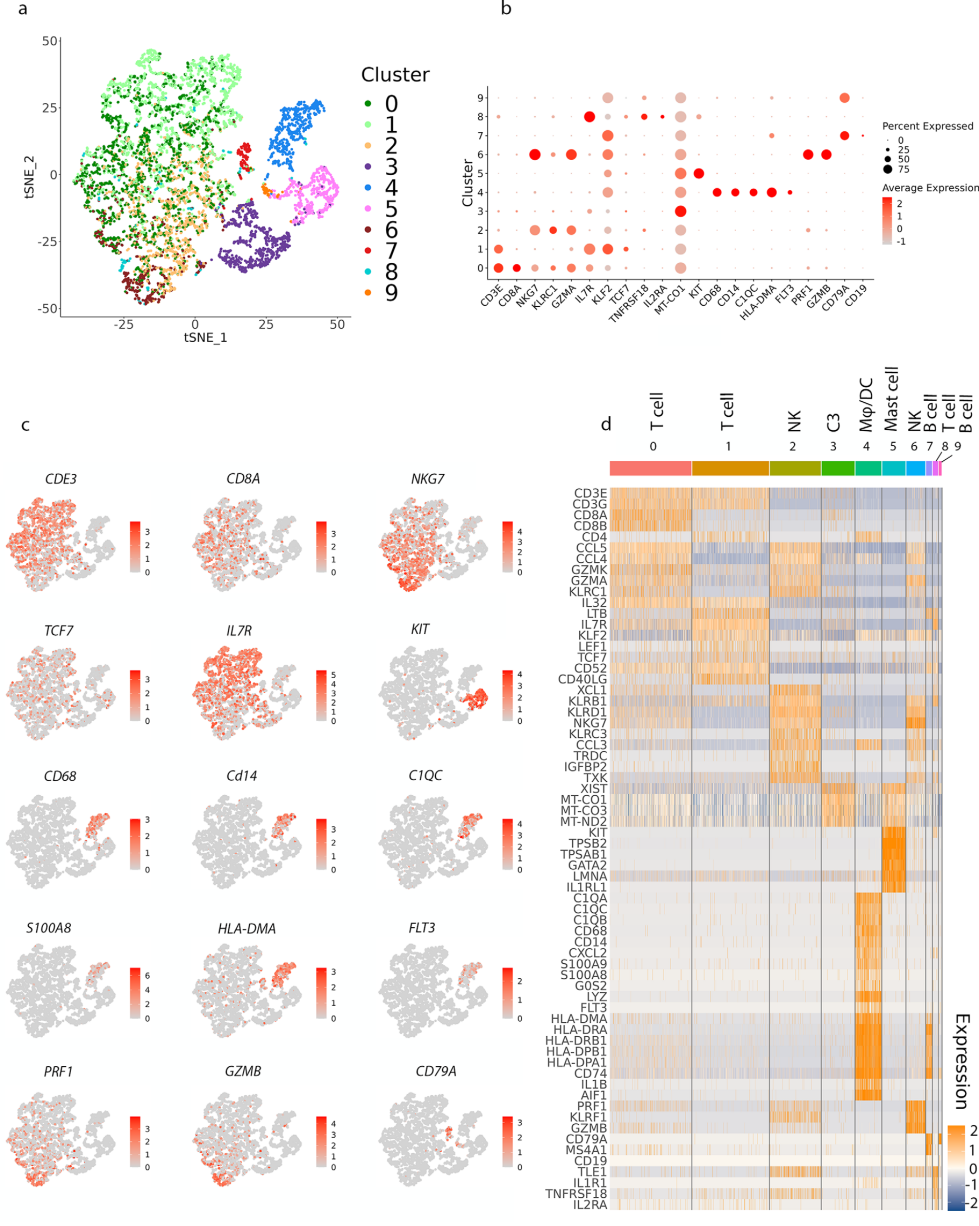
**Identifying immune cell types in the human choroid based on differentially expressed genes.** (**a**) t-SNE plot of 6501 immune cells (Cd45+; *n* = 1 donor) showing the 10 immune cell types that were identified via unsupervised clustering. (**b**) Dot plots showing the expression of cell type-specific genes, with the dot size representing the percentage of cells expressing the gene and the color representing its average expression within a cluster. (**c**) t-SNE maps showing the expression of key marker genes for the immune cell populations that identified in **b**. *Grey* indicates low expression and *red* indicates high expression. The range of log_2_ normalized counts are shown to the right of each plot. (**d**) Heatmap of normalized expression for selected genes in each cluster. Range of log_2_ normalized counts are shown to the right of heatmap. *Blue* indicates low expression and *orange* indicates high expression.

Having established a transcriptomic profile of immune cells within the mouse choroid and leptomeninges, we next sought to determine if similar populations were present in human tissues. We therefore performed scRNA-seq on immune cells (CD45^+^) isolated from the choroid of a 45-year-old male eye donor. Unsupervised clustering and t-SNE projections were performed on 6501 cells sequenced from the human choroid (70,322 mean reads per cell; Fig. 7a). Clusters were identified based on cell-specific gene expression profiles. Similar to the mouse choroid, the human choroid contained various immune cell types including B cells, T cells, DC, MC/Mφ, NK cells, and mast cells (Figs. 7b–d).

T cells were the predominant immune cell cluster identified in the human choroid. However, this may not accurately represent the composition of immune cells in situ, as (unlike the mouse choroid) it was not possible to exclude leukocytes within the vasculature from the human choroid prior to tissue processing. Three T cell clusters (cluster 0, cluster 1, and cluster 8) were identified in the human choroid. Cluster 8 was a small cluster that expressed *IL7R*, *KLRB1*, *TXK*, *IL1R1*, *TNFRSF18*, *TLE1*, and *IL2RA.* T cell cluster 0 expressed genes consistent with cytotoxic CD8^+^ T cells (*CD8A*, *CD8B1*, *NKG7*, *GZMA*, and *KLRC1*), whereas T cell cluster 1 expressed genes consistent with naïve T cells and CD4^+^ effector memory T cells, such as IL*7R*, *KLF2*, *TCF7*, and *LEF1* and *CD4*. Although several MC/Mφ and DC clusters were identified in the mouse choroid, only one cluster (cluster 4) expressed genes characteristic of MC/Mφ (*CD14* and *CD68*) and DCs (*FLT3*) in the human choroid (Figs. 7b–d). This cluster was also characterized by expression of *HLA-DMA*, *HLA-DRA*, *HLA-DRB1*, *HLA-DPB1*, *HLA-DPA1*, and *CD74*, suggesting that cells within this cluster likely function in antigen processing and presentation. It is possible that with increased sequencing depth, this cluster may resolve into individual MC/Mφ and DC populations as observed in the mouse choroid. In addition, *S100A9* and *S100A8* were upregulated in a part of this cluster. Cluster 3 (C3) had high level of mitochondrial gene expression such as *MT-CO1* and *MT-CO2* which could be a result of high fraction of apoptotic or lysing cells in the sample given the delay between donor time of death and sample processing.

Overall, these findings demonstrated that the human choroid contained MC/Mφ/DCs, NK cells, mast cells, T cells, and B cells (see Fig. 7d). However, due to differences in cell numbers sequenced, sequencing depth, and inability to perfuse human tissue prior to processing, direct comparison of clusters and their transcriptomic signatures was not possible between human and mouse tissues.

## Discussion

This study used scRNA-seq to generate a transcriptomic cell profile of immune cells within the healthy mouse choroid and leptomeninges. Multiple immune cell types were identified in these homologous CNS bordering tissues, with some clusters common to both tissues and others exhibiting tissue-specific gene expression signatures. Myeloid cells were the predominant immune cells detected within mouse choroid and leptomeninges, consistent with previous literature demonstrating populations of macrophages and DCs within these tissues.^9^^,^^10^^,^^34^ An important outcome of this study is the identification of unique sets of DEGs for each of the identified choroidal and leptomeningeal macrophage and DC clusters, which may be useful for defining cell-specific markers for these subtypes. This may enable future studies to map the spatial distribution of macrophage and DC subtypes and expand our understanding of their role in healthy conditions and during inflammatory diseases of the CNS.

We did identify some similarities between selected myeloid clusters in mouse choroid and leptomeninges. Leptomeningeal MC/Mφ cluster 3 and choroidal MC/Mφ cluster 2 appeared to be equivalent macrophage populations, sharing common top upregulated DEGs and over-represented GO terms. However, in contrast to leptomeninges, the choroid did not contain a cluster that resembled *Cd163^+^* Mφs. Of the three MC/Mφ clusters identified in leptomeninges, leptomeningeal MC/Mφ cluster 9 expressed genes consistent with *Cd163^+^* Mφs, which were previously reported in the subdural meninges.^9^ Interestingly, this cluster showed DEGs related to amoeboid migration, which was unique among leptomeningeal macrophage clusters. The mobility of immune cells within the meningeal spaces, such as T cells and antigen presenting cells, and their migration to other sites has been demonstrated previously.^35^^–^^38^ Our data potentially extend these findings to include leptomeningeal macrophages, although further investigation is warranted. The differences in gene expression observed between leptomeningeal and choroidal MC/Mφ clusters is consistent with previous studies demonstrating differences in the ontogenies of macrophages within the subdural meninges and choroid. Whereas subdural meningeal macrophages are prenatally derived, long-lived cells that are maintained through in situ self-renewal,^8^ choroidal macrophages are short-lived monocyte-derived cells that are replenished from the bone marrow.^39^

Additionally, both the choroid and leptomeninges contained a cDC2 cluster with similar transcriptomic signatures. The choroid also contained cDC1, DC-prolif, and Neu-like MC clusters, which were absent in leptomeninges. An unanswered research question is why DCs are present in the choroid in the absence of traditional lymphatics. One hypothesis is that choroidal antigen presenting cells could migrate to lymph nodes via lymphatic vessels in the optic nerve sheath^40^^,^^41^; however, the pathways of DC migration from the choroid have not been demonstrated. It is possible that a greater number of myeloid clusters within the choroid may be required for enhanced surveillance due to the different vascular properties of this tissue (fenestrated blood vessels) compared to the leptomeninges (non-fenestrated pial blood vessels).^2^^,^^42^ A limitation of our study though, is that a larger number of cells were sequenced from the choroid (2125 cells) compared to leptomeninges (1418 cells). It is therefore possible that the reduced number of cell types identified in leptomeninges compared to choroid is due to the lower number of immune cells sequenced. Despite these limitations, an advantage of our study is the high sequencing depth achieved for mouse choroid and leptomeninges, which enables higher accuracy in determining the true transcriptional profile of cells.^43^

In leptomeninges, we identified a distinct cluster of neutrophils. A previous study reported that the dura mater and pia mater contained a large population of neutrophils, and that most of these cells were bona fide extravascular cells and not contaminants from the circulation or lumen of blood vessels.^44^ Although the role of neutrophils in neuroinflammation has been investigated,^45^ their function in the healthy meninges is not well understood. It is hypothesized that neutrophils residing within the tissues need to be regulated to avoid degranulation and prevent CNS damage.^46^ Although we did identify a putative Neu-like MC cluster in the mouse choroid, classical neutrophils were exclusively identified in leptomeninges and not the choroid. Neu-like MC are granulocyte monocyte progenitor (GMP)-derived neutrophil-like monocytes that produce proinflammatory cytokines such as IL-1β.^28^^,^^29^ Recently, Chen et al. found a unique Neu-like MC cluster, similar to that described in our study, in an anti-glomerular basement membrane crescentic glomerulonephritis mouse model.^29^ To the best of our knowledge, this is the first study to identify a putative Neu-like MC population in the choroid. Given that Neu-like MCs play a role in disease in other organ systems, future studies of choroidal Neu-like MCs may reveal a novel role for these cells in ocular inflammation, although further work is required to validate the identity and function of these cells.

We identified B cell, T cell, and NK cell clusters within both the choroid and leptomeninges using scRNA-seq and demonstrated the presence of B cell and T cells in these tissues using immunohistochemistry. These findings are consistent with a previous scRNA-seq study that showed B cell, T cell, and NK cell populations within the healthy mouse dura mater, enriched subdural meninges, and choroid plexus.^9^ T cells were also identified in the RPE/choroid using single cell RNA sequencing in the mouse^47^ and humans.^48^ When compared to previous studies of the subdural meninges,^9^ our data showed a higher proportion of B and T cells within leptomeninges. These differences may be explained by differences in experimental conditions, for example, such as different mouse sexes used between these two studies or differences in animal housing environments. We also cannot exclude the possibility that a small portion of lymphocytes identified in our study may be peripheral contaminants, as previously mentioned. Shafflick et al.^32^ demonstrated that following perfusion, peripheral blood leukocytes represented 3.22% of CD45+ cells within rat pia mater-enriched subdural meninges. However, as the clusters identified in our study demonstrated tissue-specific gene expression signatures, they are unlikely to be contaminating peripheral blood cells from the vasculature. Future studies assessing the origin and density of lymphocyte populations within these tissues are required to validate the in situ distribution and fate of B and T cell clusters.

Within the CNS and its bordering tissues, most studies have focused on microglia, macrophages, and T cells, whereas little is known about B cells. Recent scRNA-seq studies have substantiated the concept of long-term residence and local development of B cells within the meninges.^32^^,^^49^^,^^50^ Specifically, these studies have revealed that meningeal B cells derive from calvarial bone marrow and primarily congregate near the dural sinuses, where endothelial cells express crucial niche factors conducive to B cell development.^50^ Here, we demonstrated that B cells are also present within mouse leptomeninges and choroid, as well as the human choroid. Whether leptomeningeal B cells are derived from the calvarial-meningeal pathway of B cell development,^49^ or from systemic circulation remains to be determined. Furthermore, the origin of choroidal B cells remains to be investigated.

Comparison of B cell clusters within the healthy mouse choroid and leptomeninges revealed that *Cxcr4* is highly expressed by the choroidal B cell cluster but not the leptomeningeal B cell cluster. *Cxcr4* plays a crucial role in regulating the homeostasis of B cells and humoral immunity, as inactivation of Cxcr4 has been linked to decreased numbers of B cells and defective T cell-independent responses in the peritoneal cavity.^51^ Additionally, the choroidal B cell cluster differentially expressed *Cd69*, a molecule that triggers B cell activation and the maturation of DCs, via direct cellular contact, that have an amplified capacity to promote Th2 responses.^52^ Genes that were enriched in the leptomeningeal B cell cluster (but not expressed by the choroidal B cell cluster) included *Id3*, *Dok3*, and *Slamf6*. *Id3*, a transient inhibitor of E protein activity,^53^ is known to be expressed by naïve B cells and plays a role in the ability of activated B cells to undergo expansion, class switching recombination and germinal center development.^54^ Upon exposure to antigens, downregulation of *Id3* results in the release of E2A and E2-2 proteins that are essential for antigen-induced B cell differentiation.^55^ *Dok3* is an adapter molecule involved in the recruitment of inhibitory molecules, which is suggested to play a role in negative regulation of immunoreceptor signaling in B cells and macrophages.^56^ Finally, *Slamf6* is involved in the interaction between naïve B and T cells. This interaction results in upregulation of T cell cytokine migration inhibitory factor, leading to augmented expression of its receptor CD74 on B cells, thereby enhancing B cell survival.^57^ The variations in gene expression between leptomeningeal and choroidal B cells suggest nuanced molecular signatures underlining the specialized functions of B cell clusters in distinct anatomic locations within the CNS supporting tissues.

We also performed scRNA-seq of immune cells isolated from human choroid-RPE. Similar to the mouse choroid, the human choroid contained DCs, macrophages, NK cells, mast cells, T cells, and B cells. Similar immune cell populations were identified in human postmortem choroid-RPE tissue by Yu et al.^58^ Together, these findings highlight the diverse immunological landscape of the human choroid. A caveat of our study is that further interpretation of the human scRNA-seq data are limited due to cells being obtained from only one donor, a lack of perfusion to remove circulating leukocytes from the vasculature of the human choroid, and the time delay between tissue retrieval by tissue bank personnel and processing for scRNA-seq (17 hours).

In conclusion, scRNA-seq revealed that mouse choroid and leptomeninges contained similar immune cell types; however, tissue-specific immune cell clusters and gene expression signatures were also characterized. These differences may be explained by the different vascular properties of the choroid (leaky fenestrated blood vessels) compared to the leptomeninges (non-fenestrated pial blood vessels with tight junctions).^2^^,^^42^ However, recent studies demonstrating a role for immune cells in maintaining tissue homeostasis may indicate that the differences in immune environments between the choroid and leptomeninges represent functional specializations to support the underlying retina and brain, respectively. Overall, our findings serve as a base for understanding the diversity of immune cells in the CNS bordering tissues. Although further research is required to validate the identified immune cell clusters, their spatial distribution, and function.

## Supplementary Material

Supplement 1
