# Supplementary material for: Tissue-Specific Immune Transcriptional Signatures in the Bordering Tissues of the Mouse Retina and Brain
Source: Invest Ophthalmol Vis Sci. 2024 Oct 28;65(12):42. doi: 10.1167/iovs.65.12.42 (PMC11514940; doi:10.1167/iovs.65.12.42)
Supplement: Supplement 1 [file iovs-65-12-42_s001.pdf]

Supplementary Table 1. Key genes used to putatively identify immune cell clusters.

Cell type	Signature genes
**T cells**	*Cd3e*
**B cells**	*Cd19, Cd79a*
**Microglia**	*Tmem119, P2ry12*
**Monocytes/ macrophages/microglia**	*Cd68, Cd14*
**Dendritic cells**	*Flt3*
**DC1**	*Clec9a*
**DC2**	*Cd209a*
**Neutrophils**	*Ngp*
**NK cells**	*Klrb1c*
**Mast cells**	*Kit*

Supplementary Table 2. Comparison of choroidal and leptomeningeal T cell cluster DEGs with defining gene signatures of T cell subsets within peripheral blood.

**Mouse peripheral blood leukocytes (Teo *et al*. 2023)[33]**		**Current study**
**T cell subtype**	**Defining genes in peripheral blood of young C57Bl/6 mice**	**Expressed by T cell clusters in choroid and leptomeninges?**
Naïve T cells	*Sell*	Not differentially expressed
Memory T cells	*Klrb1c, Id2, Cd44*	*Id2* differentially expressed in choroid cluster 3, leptomeninges cluster 0 and leptomeninges cluster 6.<br><br>*Klrb1c* and *Cd44* not differentially expressed.
Cytotoxic T cells	*Gzma*	Not differentially expressed
